# Language proficiency drives listening-related fatigue in those who communicate in English as their second language

**DOI:** 10.3758/s13423-026-03005-3

**Published:** 2026-09-17

**Authors:** Ronan McGarrigle, Jelena Havelka, Angela de Bruin

**Affiliations:** 1 School of Psychology, University of Leeds, Leeds, LS2 9JT UK; 2 Department of Psychology, University of York, York, UK

**Keywords:** Listening-related fatigue, Effortful listening, Speech perception, Bilingualism, L2 listening, Language proficiency

## Abstract

Individuals who listen to speech in their second language (‘L2 listeners’) are disproportionately impacted by adverse acoustic conditions; a phenomenon coined the ‘non-native speech-in-noise disadvantage’. Evidence also suggests that L2 listening taxes cognitive resources to a greater extent than in L1 listeners. Very little, however, is known about the longer-term consequences of this sustained cognitive engagement. Listening-related fatigue refers to the daily life subjective experience of extreme tiredness from effortful listening. The current study recruited a large sample of L2-English listeners (*N* = 256) to explore factors that predict listening-related fatigue. Approximately half of this sample (*n* = 126) reported an Indo-European (IE) language (e.g., Italian) as their first language, whereas the other half (*n* = 130) reported a non-Indo-European (non-IE) language (e.g., Vietnamese) as their first language. Non-IE L2 listeners reported higher overall listening-related fatigue than both IE L2 listeners and a group of monolingual English-speakers (L1-English). Self-rated proficiency and objective vocabulary scores were significant predictors of L2 listening-related fatigue and they moderated the effect of L2 use on listening-related fatigue; individuals who reported increased L2 use were more likely to experience increased fatigue, but only if their self-rated proficiency was low or moderate or their vocabulary score was low. Exploratory mediation analysis suggested that the effect of the L2 listener group (IE vs. non-IE) on listening-related fatigue was attributable to lower self-rated proficiency and objective vocabulary in the non-IE L2 group. To conclude, language proficiency appears to be a strong determinant of listening-related fatigue in L2-English listeners. When L2-English proficiency is low, increased L2-English usage is associated with heightened listening-related fatigue.

**Supplementary Information:**

The online version contains supplementary material available at https://doi.org/10.3758/s13423-026-03005-3.

## Introduction

We live in a world where it is increasingly common to encounter a multitude of languages and a range of environmental noises and attentional distractions (e.g., smart phone use). In multilingual environments with competing noise and distractions, L2 listening is frequently effortful (Lecumberri et al., 2010). Slower access to lexical representations causes downstream effects on the ability to follow typically fast-paced conversations, which often involve multiple interlocutors (Scharenborg & van Os, 2019). The difficulty of L2 listening is exacerbated when processing speech in everyday noise (Bsharat-Maalouf et al., 2023; Lecumberri et al., 2010).

The challenge of L2 listening may not always surface as a decline in behavioural performance. Borghini and Hazan (2018) used pupillometry (an eye-tracking technique measuring changes in pupil dilation) to assess listening effort in a group of L1 (English monolingual) and L2 (Italian-English) listeners. Both groups performed a speech recognition task in quiet and levels of background noise that were individually adapted to approximate a given performance level. Pupil dilation responses were larger (suggesting increased effort) in the L2 group across all listening conditions (Borghini & Hazan, 2018). Thus, even when there is no discernible effect on performance (i.e., speech is recognised), L2 listening incurs a processing cost. Recent pupillometry research investigating multilingual effort in Arabic-Hebrew-English speakers suggested that listening effort is modulated by language experience, with greater experience associated with reduced effort (Bsharat-Maalouf et al., 2025). Thus, there is experimental evidence for increased speech-processing costs in L2 listeners, particularly in the presence of noise. However, the longer-term consequences of routine exposure to everyday L2 listening scenarios remains unknown.

Listening-related fatigue refers to feelings of extreme tiredness from repeated and/or sustained effortful listening, a common unwanted feature of everyday life for individuals with hearing loss (Hornsby et al., 2021). However, listening-related fatigue is also reported in non-clinical populations, including individuals with heightened sensory-processing sensitivity or mood disturbance (McGarrigle & Mattys, 2023; McGarrigle et al., 2021). Although speech-processing difficulties in L2 and hearing-impaired listeners arise from different sources, there is likely to be some overlap in the net consequences of these perceptual challenges, including experiences of stress, frustration, effort, and fatigue.

The Data-Resource-Language framework (Mattys et al., 2025) characterises challenges to speech perception arising from either an impoverished auditory system (characteristic of a hearing loss) or suboptimal knowledge of the spoken language (characteristic of L2 listening). The Data-Resource-Language framework posits that reduced support from linguistic processes (e.g., vocabulary knowledge), considered a hallmark of L2 listening, results in more widespread engagement of cognitive resources (e.g., working memory) across a range of everyday listening conditions. This limited linguistic support, and increased resource engagement, should be most pronounced in individuals with low proficiency. The subjective experience of effort during communication is likely to precede more chronic experiences of fatigue (Pichora-Fuller et al., 2016). Therefore, the extent to which L2 listeners routinely experience listening-related fatigue should be driven primarily by their L2 proficiency.

Factors beyond L2 proficiency may also explain variability in listening-related fatigue. Switching between multiple languages might require additional effort, compared to processing words in one language. Alternating between languages requires not only activation of words in the to-be-attended language but also inhibition of words in the language that should no longer be used (‘language control’; Green & Abutalebi, 2013). Evidence for the role of inhibition comes predominantly from the production literature (e.g., Coumel et al., 2024; Declerck et al., 2019). However, switching costs have also been observed in the literature looking at processing of *spoken* words (e.g., Coumel et al., 2024; Fernandez et al., 2019; Olson, 2017), suggesting that bilinguals use language control during comprehension too. During comprehension, competition between the two languages might be reduced and less language control (inhibition) needed. In line with the argument that inhibition is applied relative to the strength of the language that needs to be suppressed, this control might be especially critical to apply over the L1 while listening to the L2 (e.g., Coumel et al., 2024; Fernandez et al., 2019). The amount of control might be further exacerbated in contexts where two languages are present but must be used separately (cf., ‘dual language’ environments; Hartanto & Yang, 2020).

The impact of using an L2 could also depend on the linguistic similarity with the L1. For example, poorer L2-English language outcomes have been found in individuals whose L1 is more distant from English (e.g., Japanese) than those with a more typologically similar L1 (e.g., French; Booth et al., 2018).^1^ Typological distance between L1 and L2 has even been found to negatively influence learning of a third language (Schepens et al., 2016). L2 listening may therefore come at a cognitive cost which is exacerbated with typological distance from L1. Another factor that may influence listening-related fatigue is L2 usage frequency. More frequent L2 use is likely to be associated with higher proficiency (Schmidtke, 2016) and thus lower effort and fatigue (Bsharat-Maalouf et al., 2025). However, because of sustained and/or repeated exposure, it could be argued that listening-related fatigue is *most* plausible in cases where an individual must use their L2 on a more frequent basis. A positive association between L2 usage and listening-related fatigue might therefore emerge only in individuals with low levels of L2-English proficiency.

The current study aimed to explore prevalence of listening-related fatigue in L2-English listeners and identify sources of variability in this population. First, we examined the impact of language distance by comparing overall listening-related fatigue in three distinct groups: (1) L2-English listeners whose first language is Indo-European (L2_IE); (2) L2-English listeners whose first language is non-Indo-European (L2_nonIE); and (3) monolingual English-speakers (L1_English). We examined predictors of listening-related fatigue in L2-English listeners, including self-rated L2-English proficiency and an objective vocabulary measure, frequency of L2 usage, and frequency of language switching in dual-language contexts (where bilinguals likely require most language control). We included both vocabulary (LexTALE) and self-rated proficiency measures for a comprehensive picture of overall L2 English proficiency. We made the following predictions:

- H1. Monolingual English-speakers will report less listening-related fatigue than the L2_IE group, who, in turn, will report less fatigue than the L2_nonIE group, supporting an effect of L1/L2 language distance on listening-related fatigue (Booth et al., 2018).
- H2. Self-rated L2 proficiency and objective vocabulary scores will significantly negatively predict listening-related fatigue (Mattys et al., 2025).
- H3. Frequency of language switching in dual-language contexts will be positively associated with listening-related fatigue, reflecting their increased inhibitory control demands (Coumel et al., 2024).
- H4. L2 proficiency (self-rated proficiency and objective vocabulary score) will moderate the effect of L2 usage frequency on listening-related fatigue, such that increased L2 use will be associated with higher fatigue scores in individuals with low L2 proficiency only (Bsharat-Maalouf et al., 2025; Schmidtke, 2016).

## Method

Methodological plans and hypotheses for this study were pre-registered (https://osf.io/wj59n/registrations). Raw and summary data can be accessed from our Open Science Framework (OSF) project homepage (https://osf.io/wj59n/overview).

### Participants

We recruited 427 participants, aged 18–50 years (*M* = 32.24 years, *SD* = 7.78). Of these participants, 237 selected ‘Female’ as their gender, 186 selected ‘Male’, three selected ‘Other’, and one selected ‘Prefer not to say’. All participants were recruited via the online recruitment platform Prolific (prolific.co) and financially compensated for their time at a rate of £9 p/h. The following initial eligibility criteria were applied on Prolific for all three groups, based on self-reports: (1) Based in UK, USA, or Canada; (2) aged between 18 and 50 years; (2) had no hearing difficulties; (3) were not currently wearing a cochlear implant; (4) had no known language-related disorders; (5) were fluent in English; and (6) had no long-term health condition/disability.

The following additional criteria stored on the Prolific database were used to assign participants into one of the three participant groups: (1) Monolingual L1_English; (2) L2_IE; and (3) L2_nonIE. Participants in the ‘L1_English’ group were pre-selected using the following Prolific screeners: (1) First language identified as ‘English’; (2) self-identified as ‘English-speaking monolingual (I only know English)’; and (3) Responded ‘I was raised with my native language only’ to the question ‘Were you raised monolingual?’. In addition to the four customised eligibility questions, participants in this group were also asked to confirm in the study survey that they were ‘a monolingual English speaker (i.e., you are not fluent in any language other than English)’, which all 86 participants verified. Participants in the ‘L2_IE’ group were identified as those who listed any of the following as their first language: Danish, Dutch, French, German, Italian, Norwegian, Portuguese, and Swedish. Participants in the ‘L2_non-IE’ group were identified as those who listed any of the following as their first language: Burmese, Chinese, Indonesian, Korean, Nepali, Thai, Tibetan, and Vietnamese.

**Fig. 1:**
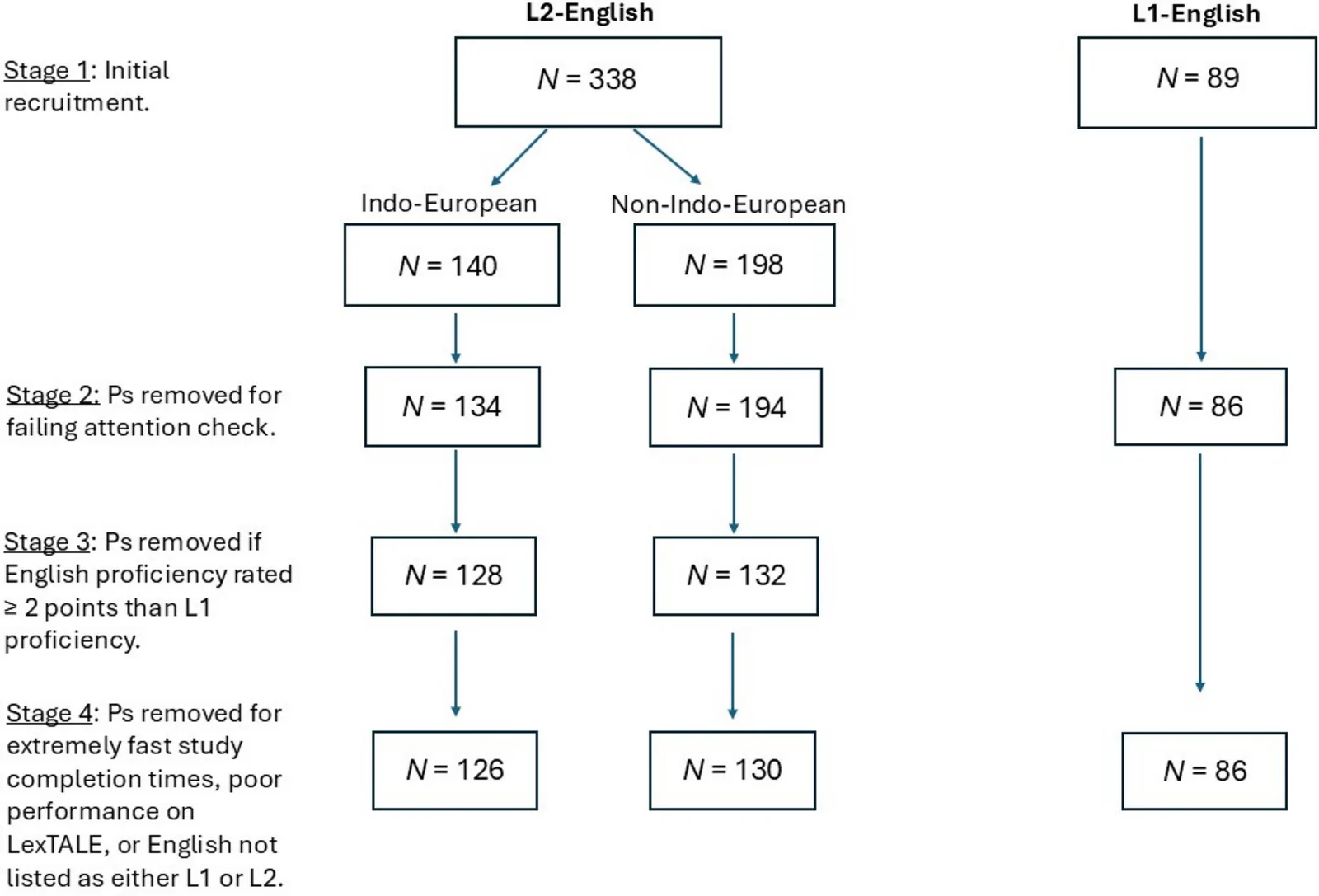
Schematic representation showing participant numbers at each stage of the screening procedure

Based on a power analysis in G*Power (Faul et al., 2009), we calculated that 250 L2-English listeners would provide 80% power to detect a small–medium effect (Cohen’s *f* ^2^ = 0.06) in multiple regression with seven predictors, and a smaller–medium effect (Cohen’s *f* ^2^ = 0.04) in moderation analysis with two moderators (self-rated proficiency and objective vocabulary score) with an alpha error probability of 0.05. We recruited a total of 338 L2-English listeners, along with an additional 89 monolingual controls. Of the 427 participants, 13 failed the attention check^2^ (see details in ‘Vanderbilt Fatigue Scale’ *Stimuli and task procedure* section below) and were removed from the analyses. Of the remaining 328 L2-English listeners, 68 were excluded for reporting an English understanding proficiency score of ≥ 2 points higher than their L1 understanding proficiency score (meaning that in terms of proficiency, English was likely not their L2). Finally, an additional four L2-English listeners were removed from the analyses: three for completing the experiment exceptionally fast (< 25% of median completion time) and providing uninterpretable responses (e.g., responding with a number to a question asking them to indicate their ‘Language A’), and one whose performance on the LexTALE task (a behavioural measure of L2 English vocabulary, described below) was 6.26 *SD*s below the mean (scoring just 6% accuracy), suggesting either inattention or very low English proficiency. Figure 1 provides a schematic representation of the screening process and participant exclusions at each stage. A total of 342 participants (256 L2-English listeners) were entered into the analyses. Table 1 shows the demographic and language characteristics of each group entered into the analyses. This study was granted ethics approval by the School of Psychology Research Ethics Committee at University of Leeds (Ref: PSCETHS-1085).

**Table 1:** Demographic and language use characteristics of participants entered into the analyses

	Group		
	L1 English	L2 IE	L2 nonIE
*N*	86	126	130
Age in years (*M, SD*)	33.51 (7.75)	33.43 (7.36)	31.30 (7.65)
Gender (Female/Male/Other/Undisclosed)	41/44/0/1	79/46/1/0	77/52/1/0
Age (in years) of onset English acquisition (*M*, *SD*)	/	8.86 (3.99)	6.52 (3.75)
L2 self-rated language proficiency (1–10)	/	8.91 (0.86)	7.99 (1.48)
LexTALE vocabulary score % (0–100)	/	86.5 (9.8)	79.8 (11.7)
Language use overall (1 = all L1, 5 = all L2)	/	3.25 (0.78)	3.05 (0.84)
Language use in formal (work/education) contexts (1 = all L1, 5 = all L2)	/	3.80 (1.45)	3.62 (1.64)
Language entropy (0 = language use in single-language contexts, 1 = language use in dual-language contexts)	/	0.47 (0.16)	0.46 (0.17)
Single-language context usage % (0–100)	/	63.61 (29.51)	59.72 (26.14)
Dual-language switching % (0–100)	/	22.09 (22.38)	22.02 (17.18)
Dense code-switching % (0–100)	/	33.71 (28.51)	37.66 (24.47)

^*^See Analysis section for details on how each score was calculated

### General procedure

We used Gorilla Experiment Builder (www.gorilla.sc; Anwyl-Irvine et al., 2020) to design and host the experiment. Participants were recruited on Prolific and directed to Gorilla using an experiment link. On Prolific, participants were instructed to only take part in the experiment if they: (1) could complete the study on a laptop or desktop computer; (2) were aged 18–50 years; (3) did not suffer from a long-term health condition (e.g., chronic fatigue syndrome (CFS), cancer, diabetes) or language-related disorder (e.g., dyslexia); (4) were not regularly taking any prescribed medication known to cause fatigue (e.g., anti-histamines); and (5) do not currently have a hearing loss and/or wear a hearing device (e.g., hearing aid or cochlear implant). After reading the information sheet and providing consent, participants were asked to confirm that they met all of the following customised eligibility criteria: (1) You do not currently suffer from a long-term health condition which may cause chronic fatigue (e.g., CFS, cancer, diabetes); (2) You do not regularly take any prescribed medication that is known to cause fatigue (e.g., antihistamines); (3) You do not have a hearing loss and/or currently wear a hearing device (e.g., hearing aid or cochlear implant); and (4) You do not have any kind of language-related disorder (e.g., dyslexia). Participants who responded ‘No’ to any of the above screening questions were automatically rejected from the study.

All questionnaires and tasks were completed on Gorilla. First, participants completed the Vanderbilt Fatigue Scale (Hornsby et al., 2021), followed by the Language and Social Background Questionnaire (LSBQ; Anderson et al., 2018) and Interactional Contexts Questionnaire (ICQ; Hartanto & Yang, 2020). Finally, participants completed the LexTALE before being provided with an experiment debrief. In total, the study lasted approximately 15 min. Participants in the ‘monolingual L1 English’ group completed the Vanderbilt Fatigue Scale only. For this group, the total study duration was approximately 5 min.

### Stimuli and individual task procedures

#### Vanderbilt fatigue scale

The Vanderbilt Fatigue Scale was administered to measure daily life experiences of listening-related fatigue; that is, fatigue that is experienced specifically in the context of listening (Hornsby et al., 2021). This scale has been shown to have high marginal reliability (*r* =.98), adequate test–retest reliability (*r* =.60 –.69), and evidence of good construct validity across age, gender, and hearing-impaired adult populations (all differential item functioning effect sizes ≤ 0.023). The questionnaire consists of 40 items, each with 5-point Likert-type responses (0–4) with verbal anchors ranging from ‘Never/Almost never’ to ‘Always/Almost always’ or ‘Strongly Disagree’ to ‘Strongly Agree’, none of which were reverse-coded. Examples of test items include: ‘*I feel worn out from everyday listening*’ and ‘*It takes a lot of energy to listen and understand*’. Participants are given the following (verbatim) instructions; ‘*For each item, select the SINGLE response which best describes your day-to-day experiences over a typical WEEK*.’ Following Buchanan and Scofield’s (2018) recommendations for optimising quality of data collected online, we also included an attention check. In between item numbers 36 and 37, participants were asked to ‘*Please select ‘strongly agree’ to show you are paying attention to this question*’. For the attention check item, the response options provided were the same as for items 30–40 (ranging from ‘Strongly disagree’ to ‘Strongly agree’).

#### Language and Social Background Questionnaire (LSBQ)

The LSBQ was used to assess self-rated proficiency and language use across a variety of contexts (Anderson et al., 2018). Specifically, we included questions assessing self-rated proficiency for speaking, understanding, reading, and writing as well as a section on community language-use behaviour. Participants were asked to reflect on their language use in the past 6 months, with different interlocutors (e.g., with parents, siblings, friends), in different environments (e.g., home, school, work), and for different activities (e.g., reading, using social media). Two additional answer options were included: one to indicate another language aside from English or their L1 was used in that context, and one to indicate that the context did not apply to the participant (e.g., because they did not have a partner). These answer options were not included when computing the mean score.

Finally, we left out two LSBQ context questions (praying and for religious activities) as these questions are often responded to as “not applicable” and elicit unnecessary personal (religious) information. We added three questions referring to language use during speaking, writing, and understanding speech to complement the question on reading and to make sure all questions were in the same answer format (in the original LSBQ, questions about time spent speaking, writing, and understanding each language have a different answer format). The LSBQ has been shown to have moderate-substantial test–retest reliability in capturing bilingual experiences (Mann & de Bruin, 2022) and at least moderate validity relative to Ecological Momentary Assessment (de Bruin, in press). Responses on this questionnaire were analysed to compute self-rated L2 English proficiency, frequency of L2 language use, and language entropy scores (see Analysis for more specific details).

#### Interactional Contexts Questionnaire (ICQ)

The ICQ was used to assess bilingual language-switching contexts (Hartanto & Yang, 2020). In the ICQ, participants were asked to report the prevalence of a variety of bilingual interactional contexts across four situations: home, school, work, and others. Participants report the percentage of time spent in each of these places overall and, using percentages, they indicate for each of the four contexts to what extent their daily conversational exchanges took place in a single-language context (e.g., ‘*I speak only one language and rarely switch to the other language*’), a dual-language context (e.g., ‘*I often switch languages, but I rarely mix languages within an utterance*’), or a dense code-switching context (e.g., ‘*I routinely mix two or more languages within an utterance to most speakers*’). The percentages reported in each place (e.g., home, work) must sum to 100%.

#### LexTALE

LexTALE was used as a behavioural measure of English language vocabulary (Lemhöfer & Broersma, 2012). LexTALE is a 5-min vocabulary test in which participants are asked to indicate whether a string of letters presented on the screen is a word (by pressing the letter ‘L’) or not a word (by pressing the letter ‘A’). The test includes 60 items altogether (40 words and 20 non-words), preceded by three practice items. LexTALE has been shown to be a good predictor of English vocabulary knowledge and correlated with a measure of general English proficiency (Lemhöfer & Broersma, 2012).

### Analysis

All statistical analyses were conducted using JASP v0.19.3 (JASP Team, 2024). Prior to analyses in JASP, data were processed using R v.2024.12.0.467 (R Core Team, 2025).

#### Listening-related fatigue score

Total listening-related fatigue score was a summed score of the 40 items on the Vanderbilt Fatigue Scale (excluding the attention check item). Possible scores ranged from 0 to 160, with higher scores indicating increased listening-related fatigue.

#### Objective vocabulary score

LexTALE vocabulary scores were calculated following guidelines in Lemhöfer and Broersma (2012). Percentage correct scores on the LexTALE were calculated separately for words (i.e., % letter strings correctly identified as words) and non-words (i.e., % letter strings correctly identified as non-words). Total percentage scores were calculated as the mean of the words and non-words percentage scores, with higher total scores reflecting superior vocabulary.

#### Self-rated L2-English proficiency

Total self-rated proficiency was calculated as the mean score across speaking, understanding, reading, and writing items (all Likert-type responses ranging from 1 to 10) on the LSBQ (Anderson et al., 2018). A score of 1 indicates no proficiency and a score 10 suggests native proficiency.

#### L2 language use (overall)

A mean score was taken across all reported contexts on the LSBQ, with a range from 1 to 5, with higher scores indicating more frequent L2-English use.

#### L2 language use (formal)

A mean score was taken across formal (university and/or work) contexts only on the LSBQ with a range from 1 to 5. Higher scores indicated more frequent L2-English use in formal contexts.

#### Language switching indices

Analysis procedures were based on Hartanto and Yang (2020). For each participant, frequency of single-language contexts, dual-language switching, and dense code-switching across the four situations were determined by calculating averages weighted by frequency of usage in each context, using the following formulae:

$$\mathrm{S}\mathrm{i}\mathrm{n}\mathrm{g}\mathrm{l}\mathrm{e}-\mathrm{l}\mathrm{a}\mathrm{n}\mathrm{g}\mathrm{u}\mathrm{a}\mathrm{g}\mathrm{e}\, \mathrm{c}\mathrm{o}\mathrm{n}\mathrm{t}\mathrm{e}\mathrm{x}\mathrm{t}\, \mathrm{u}\mathrm{s}\mathrm{a}\mathrm{g}\mathrm{e} \,\mathrm{i}\mathrm{n}\mathrm{d}\mathrm{e}\mathrm{x}=\sum\limits_{i=1}^{4} \frac{\begin{array}{c}{P}_{i}\end{array} \mathrm{x}\, { sl}_{i}}{100}$$

$$\mathrm{D}\mathrm{u}\mathrm{a}\mathrm{l}-\mathrm{l}\mathrm{a}\mathrm{n}\mathrm{g}\mathrm{u}\mathrm{a}\mathrm{g}\mathrm{e} \,\mathrm{s}\mathrm{w}\mathrm{i}\mathrm{t}\mathrm{c}\mathrm{h}\mathrm{i}\mathrm{n}\mathrm{g} \,\mathrm{i}\mathrm{n}\mathrm{d}\mathrm{e}\mathrm{x}=\sum\limits_{i=1}^{4} \frac{\begin{array}{c}{P}_{i}\end{array} \mathrm{x}\, { dl}_{i}}{100}$$

$$\mathrm{D}\mathrm{e}\mathrm{n}\mathrm{s}\mathrm{e}\, \mathrm{c}\mathrm{o}\mathrm{d}\mathrm{e}-\mathrm{s}\mathrm{w}\mathrm{i}\mathrm{t}\mathrm{c}\mathrm{h}\mathrm{i}\mathrm{n}\mathrm{g}\, \mathrm{i}\mathrm{n}\mathrm{d}\mathrm{e}\mathrm{x}=\sum\limits_{i=1}^{4} \frac{\begin{array}{c}{P}_{i}\end{array} \mathrm{x}\, { dc}_{i}}{100}$$

where ${P}_{i}$ denotes the amount of time spent in each situation (home, school, work, or others), ${sl}_{i}$ denotes the percentage of a single-language context in a given situation,${dl}_{i}$ denotes the percentage of a dual-language context in a given situation, and ${dc}_{i}$ denotes the percentage of a dense code-switching context in a given situation. To avoid cases where context/category of language use scores did not sum to 100, we applied a correction formula which calculated each score as a relative proportion of all scores in the given context/category. For example, if A2 = time spent at home, B2 = time spent at work, C2 = time spent at school, and D2 = time spent in ‘other’ context, the time spent at home category was re-calculated as: A2/(A2 + B2 + C2 + D2)*100. Higher overall % scores represent increased prevalence of either single-language use, dual-language switching and/or dense code-switching across contexts.

#### Language entropy

Language entropy reflects the social diversity of language use in bilinguals and is often calculated as an additional measure of language switching tendencies (Gullifer & Titone, 2020). Responses to items on the language-use behaviour section of the LSBQ (ranging from 1 to 5) were computed into entropy scores. Responses indicating either ‘all L1’ or ‘all L2’ (1 or 5) were assigned a value of ‘0’ (i.e., no entropy). Following the scoring procedure recommended in Gullifer and Titone (2020), responses indicating either ‘mostly L1’ (2) or ‘mostly L2’ (4) were assigned a value of 0.811278. This was based on summing together 0.75*log_2_ (0.75) and 0.25*log_2_ (0.75),^3^then multiplying by −1 to yield a positive value. Finally, responses indicating ‘half L1/half L2’ (3) were assigned a value of ‘1’ (i.e., full entropy). An average entropy score (across all contexts) was then calculated for each participant, ranging from 0 to 1. Scores closer to 0 reflect the compartmentalised use of one language only in specific contexts, whereas scores closer to 1 reflect more time spent in dual-language contexts where both languages are used.

#### Moderation and (exploratory) mediation analysis

Moderation analyses tested the hypotheses that self-rated L2 proficiency and objective vocabulary score will moderate the effect of L2 (vs. L1) use on listening-related fatigue (H4). Exploratory mediation analyses tested the hypothesis that L2 proficiency mediates the effect of the L2 group (IE vs. nonIE) on fatigue. Both moderation and mediation analyses were conducted using the classical process model (Hayes, 2017) macro on JASP v0.19.3. For the moderation analyses, we ran two models with L2 use entered as a continuous predictor variable and total listening-related fatigue score as the outcome variable. In the first model, self-rated L2 proficiency was entered as the moderator variable, and in the second model LexTALE vocabulary score was entered as the moderator variable; 95% confidence intervals were derived from 1,000 bootstrap samples. Following the recommendations of Hayes (2017), direct and indirect effects were deemed statistically significant if bootstrap confidence intervals were either entirely above or below zero.

## Results

### Effect of group of listening-related fatigue (H1)

**Fig. 2:**
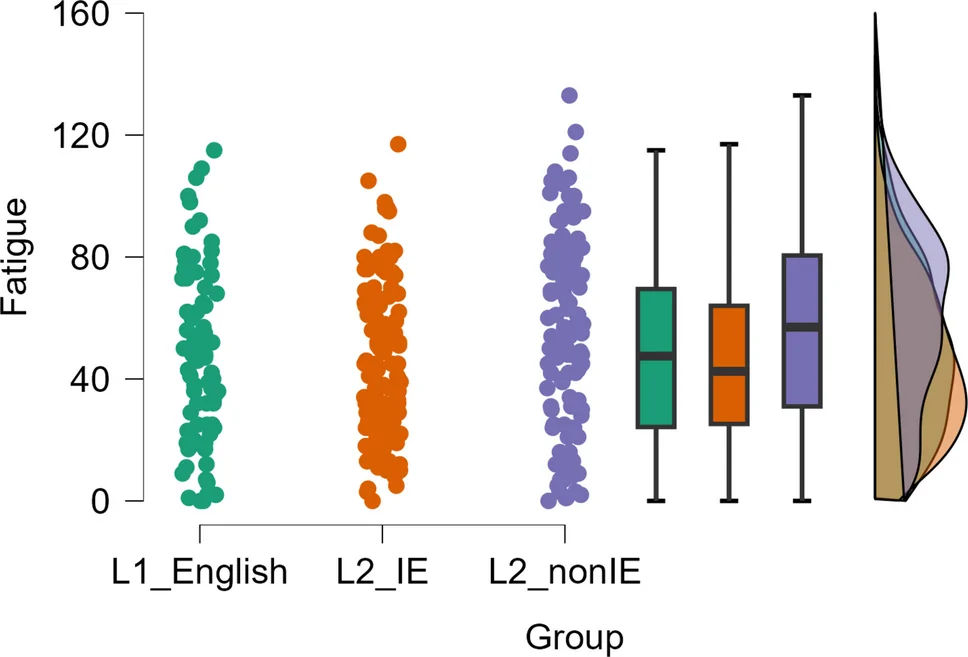
Mean total Vanderbilt Fatigue Scale (listening-related fatigue) ratings as a function of Group (L1_English, L2_IE, L2_nonIE)

Figure 2 displays mean total fatigue ratings as a function of Group. A one-way independent groups ANOVA (type 3 sum of squares) revealed a main effect of Group (*F*(_2, 340_) = 5.91, *p* =.003, partial η^2^ =.03). Post hoc comparisons using Tukey-corrected *p* values revealed that mean fatigue scores were significantly higher in the L2_nonIE group (*M* = 56.54, *SD* = 31.02) than in the L2_IE group (*M* = 44.87, *SD* = 25.35), *t* = 3.30, *p* =.003. No significant differences were found in mean fatigue scores between the L1_English group (*M* = 44.61, *SD* = 25.42) and either the L2_nonIE group (*t* = 2.32, *p* =.06) or the L2_IE group (*t* = 0.65, *p* =.79).

### Multiple regression examining predictors of listening-related fatigue in L2 listeners (H2 and H3)

Table 2 displays the correlations between all variables in the regression analysis. Due to potential multicollinearity between predictors (*r*s ≥.8), we removed L2 use (formal) and dense code-switching from the final model. We conducted a multiple linear regression analysis to examine the remaining five predictors of listening-related fatigue. Overall, the model explained 12.8% of the variance and provided a significantly better fit than an intercept-only model, *F*(5, 255) = 8.51, *p* <.001. Holding all other model predictors constant, both LexTALE vocabulary score and self-rated L2 proficiency were significant predictors of listening-related fatigue, *t*(255) = 3.54, β = -.22, *p* <.001 and *t*(255) = 4.07, β = -.27, *p* <.001, respectively. L2 use was also a significant positive predictor of listening-related fatigue, *t*(255) = 3.34, β =.22, *p* <.001. However, neither dual-language switching nor entropy significantly predicted listening-related fatigue, *t*(255) = −0.11, β = -.007, *p* =.91 and *t*(255) = 0.31, β =.02, *p* =.75, respectively.

**Table 2:** Pearson’s correlation coefficients for all variables

Variable	Fatigue	LexTALE	Self-rated proficiency	L2 Use (overall)	L2 Use (formal)	Entropy	Dual switching	Dense switching
Fatigue	**-**							
LexTALE	-.27^***^	**-**						
Self-rated Proficiency	-.21^***^	.12	**-**					
L2 Use (overall)	.14^*^	-.12	.40^***^	**-**				
L2 Use (formal)	.15^*^	-.18^**^	.34^***^	.86^***^	**-**			
Entropy	.02	-.19^**^	.10	-.08	.02	-		
Dual Switching	.03	-.12	.04	.07	.03	.26^***^	**-**	
Dense Switching	.04	-.16^**^	.06	.06	.02	.27^***^	.80^***^	**-**

^*^p <.05, ^**^p <.01, ^***^p <.001

### Moderation analyses (H4)

We ran two moderation models: one with self-rated L2 proficiency as the moderator (model 1), and one with LexTALE vocabulary score as the moderator variable (model 2). In each case, L2 Use (overall) was the IV and listening-related fatigue was the DV. In model 1, we found evidence that self-rated L2 proficiency moderated the effect of L2 use on listening-related fatigue (estimate = −4.20, *p* =.003, bootstrap CIs: −7.03 to −1.41). In other words, the effect of L2 use on listening-related fatigue differed as a function of self-rated proficiency score. To probe this conditional effect, we generated estimates of the conditional effect of L2 use on listening-related fatigue for values of self-rated L2 proficiency on the 16th percentile (i.e., ‘low’), 50th percentile (i.e., ‘moderate’), and the 84th percentile (i.e., ‘high’). Overall, we found a significant positive effect of L2 use on listening-related fatigue for individuals with low and moderate (*p*s <.001) self-rated L2 proficiency, but not for individuals with high self-rated L2 proficiency (*p* >.05). Table 3 displays the estimates and bootstrap CIs for each conditional effect.

**Table 3:** Conditional effect coefficients for model 1 (moderation analysis with ‘self-rated L2 proficiency’ as the moderator variable)

								95% CIs	
			Self-rated L2 proficiency	Estimate	*SE*	z-value	*p*	Lower	Upper
L2 Use	→	Fatigue	Low	13.52	2.68	5.05	<.001	8.87	18.90
L2 Use	→	Fatigue	Moderate	7.76	2.29	3.39	<.001	3.37	12.51
L2 Use	→	Fatigue	High	3.01	3.03	1.00	.32	−2.97	9.50

Confidence intervals (CIs) are percentile bootstrapped. Standard errors, *z* -values and *p* -values are based on the delta method. ‘Low’, ‘Moderate’, and ‘High’ self-rated rated L2 proficiency scores represent scores on the 16th, 50th, and 84th percentiles, respectively

In model 2, we found evidence that LexTALE vocabulary score moderated the effect of L2 use on listening-related fatigue (estimate = −73.05, *p* =.02, bootstrap CIs: −138.70 to −11.71). The effect of L2 use on listening-related fatigue differed as a function of vocabulary score. To probe this conditional effect, we generated estimates of the conditional effect of L2 use on listening-related fatigue for values of vocabulary score on the 16th percentile (i.e., ‘low’), 50th percentile (i.e., ‘moderate’), and the 84th percentile (i.e., ‘high’). Overall, we found a significant positive effect of L2 use on listening-related fatigue for individuals with low vocabulary scores (*p* =.002), but not for individuals with moderate or high vocabulary scores (*p*s >.05). Table 4 displays the estimates and bootstrap CIs for each conditional effect.

**Table 4:** Conditional effect coefficients for model 2 (moderation analysis with ‘LexTALE vocabulary score’ as the moderator variable)

								95% CIs	
			LexTALE vocabulary	Estimate	*SE*	z-value	*P*	Lower	Upper
L2 Use	→	Fatigue	Low	16.68	5.46	3.06	.002	6.17	27.88
L2 Use	→	Fatigue	Moderate	6.45	3.60	1.79	.07	−0.99	13.68
L2 Use	→	Fatigue	High	−0.85	5.10	−0.17	.87	−11.75	9.39

Confidence intervals (CIs) are percentile bootstrapped. Standard errors, *z* -values and *p* -values are based on the delta method. ‘Low’, ‘Moderate’, and ‘High’ LexTALE vocabulary scores represent scores on the 16th, 50th, and 84th percentiles, respectively

### Mediation analysis

**Fig. 3:**
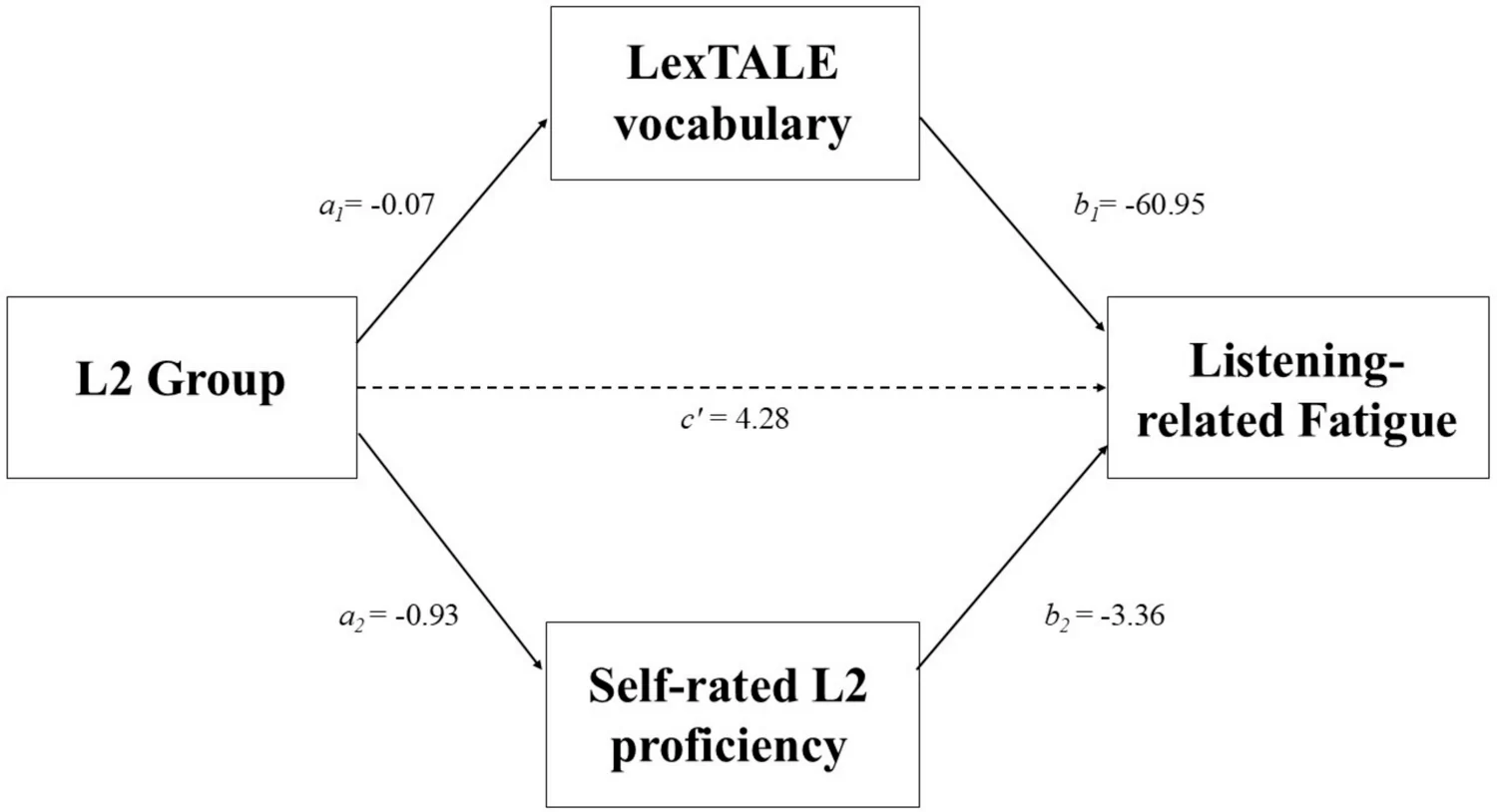
Diagram showing regression coefficients between all variables tested in the exploratory mediation model. A solid arrow connecting variables represents a significant regression coefficient (*p* <.05). A dashed arrow represents a non-significant coefficient

Exploratory mediation analysis tested the hypothesis that self-rated L2 proficiency and/or LexTALE vocabulary score mediate the effect of L2 Group (IE vs. nonIE) on listening-related fatigue (see Fig. 3). We found an indirect effect of group on listening-related fatigue via both vocabulary score and self-rated L2 proficiency. Specifically, participants in the L2_IE group performed significantly better on the vocabulary task (*p* <.001) and also reported significantly higher L2 proficiency (*p* <.001) than their nonIE counterparts. Participants who performed better on the vocabulary task and reported higher L2 proficiency scores were also significantly more likely to report lower listening-related fatigue (*p* <.001 and *p* =.02, respectively). Bootstrap confidence intervals for the indirect effect of group on listening-related fatigue via vocabulary score (*a*_*1*_*b*_*1*_ = 4.15) and self-rated L2 proficiency (*a*_*2*_*b*_*2*_ = 3.12) were both entirely above zero (1.72–6.98 and 0.60–5.52, respectively). There was no significant direct effect of group on listening-related fatigue as the bootstrap confidence intervals straddled zero (*c*′ = 4.28, bootstrap CIs: −3.32 to 12.15).

## Discussion

We examined individual differences in the experiences of listening-related fatigue amongst L2 listeners by testing the effects of L1/L2 language distance, L2 proficiency and usage frequency, and language-switching tendencies on listening-related fatigue. Our first hypothesis (H1) was partially supported. We found evidence of increased listening-related fatigue in L2-English listeners whose L1 was more typologically distinct from English (non-Indo-European) than L2-English listeners with an Indo-European L1. However, no significant differences in listening-related fatigue were found between the L1_English group and either of the L2 listener groups. We found support for our H2; both self-rated L2 proficiency and L2 vocabulary scores were significant negative predictors of listening-related fatigue. However, neither language entropy nor switching frequency were found to be significant predictors. Thus, our third hypothesis (H3) predicting an effect of language-switching tendences was not supported. Our fourth and final hypothesis (H4) was supported; both self-rated L2 proficiency and objective vocabulary score moderated the effect of L2 usage frequency on listening-related fatigue. Specifically, L2 use positively predicted listening-related fatigue, but only in those with low L2 vocabulary scores, or low-moderate self-rated L2 proficiency scores.

The non-Indo-European L2 group reported significantly higher listening-related fatigue than their Indo-European counterparts. Although there was also a numerical difference between the non-Indo-European L2 group and the monolingual English group, it was not statistically significant. Finally, no difference in listening-related fatigue was found between the Indo-European L2 group and the monolingual English group. Overall, these findings lend support to the idea that L1/L2 language distance may impact the extent to which an L2 listener experiences speech perception challenges and listening-related fatigue (Booth et al., 2018; Schepens et al., 2016). Although we did anticipate higher fatigue ratings in both L2 listener groups compared to English monolinguals, we believe that this null finding, along with the large discrepancy in listening-related fatigue between L2 listener groups, may be driven primarily by language proficiency. Notably, the Indo-European L2 group had significantly higher vocabulary and self-rated L2-English proficiency scores than their non-Indo-European counterparts (*p*s <.001; see Table 1 for *M*s/*SD*s). To test this assumption, we conducted an exploratory mediation analysis and found evidence that the effect was fully mediated by L2-English vocabulary score and self-rated L2-English proficiency (see Fig. 3). This suggests that the group difference was likely attributable to the relatively high levels of L2-English proficiency in the Indo-European L2 group which protected against the onset of listening-related fatigue. Overall, differences in listening-related fatigue between all three groups appeared to be driven most strongly by language proficiency.

Self-rated and objective markers of L2-English proficiency were the strongest predictors of L2 listening-related fatigue,^4^ with lower levels of proficiency associated with higher levels of listening-related fatigue. This finding underscores the importance of L2 proficiency in determining the likelihood (and extent) of listening-related fatigue. The Data-Resource-Language framework (Mattys et al., 2025) proposed that L2 listeners cope with the demands of everyday listening by allocating cognitive resources across a wider variety of contexts (e.g., even in relatively quiet conditions) and in a more sustained manner. Unlike monolinguals listening in their L1, the extent to which this occurs (and has repercussions including fatigue) is determined primarily by the listener’s speed of lexical access and the integrity of their lexical representations, and, thus, their L2 proficiency. The current findings therefore broadly resonate with key tenets of the Data-Resource-Language framework. Findings also support earlier empirical work on the importance of vocabulary and language proficiency in determining speech recognition performance (Schmidtke, 2016) and listening effort (Bsharat-Maalouf et al., 2025) and extends this suite of speech perception outcomes to listening-related fatigue.

Previous work highlights the cognitive demands of switching between multiple languages within a conversation (Green & Abutalebi, 2013). Research on language production highlights the importance of the linguistic environment, with less flexible dual-language use shown to increase behavioural and subjective markers of effort (de Bruin & McGarrigle, 2024). In the current study, neither dual-language switching nor a dense code-switching environment exposure predicted listening-related fatigue. We calculated an ‘entropy’ score for each listener which captures the extent to which individuals use two languages in a compartmentalised (low entropy) or flexible (high entropy) manner (Gullifer & Titone, 2020), but they also did not predict listening-related fatigue. This suggests that, while switching between languages may be cognitively costly, exposure to different types of language switching environments has relatively little impact on listening-related fatigue. While this was contrary to our expectations, it is in line with recent studies suggesting comprehension of language switches might be less cognitively demanding than production*,* potentially in part due to language comprehension eliciting less language competition (e.g., Coumel et al., 2024; Declerck et al., 2019). Thus, bilinguals might experience relatively little *listening*-related fatigue in dual-language contexts.

Finally, increased L2 usage frequency was associated with heightened reports of listening-related fatigue, but crucially this was unique to individuals with low-moderate self-rated L2-English proficiency or low L2-English vocabulary scores. For individuals with high L2 proficiency, no association was found. This suggests that increasing one’s L2 usage may come at a cost until one attains high levels of proficiency. There is an intriguing possibility that a ‘tipping point’ of language proficiency exists (e.g., between moderate-high) at which increased L2 usage no longer causes fatigue. This finding has implications for second-language learning more generally. While increasing one’s L2 usage will no doubt help to improve proficiency in the longer-term, there may also be short-term negative consequences to navigate, including listening-related fatigue. Individuals experiencing such repercussions may benefit from targeted interventions, such as listening breaks and/or counselling (Davis et al., 2021).

Study limitations include concerns about online research data quality, particularly when using largely questionnaire-based methods which may be more prone to bias. To help address some of these limitations, we followed the recommendations of Buchanan and Scofield (2018) for identifying low-quality online data which resulted in the removal of data from 85 participants in total (see Participants section for details). While the current study focused on linguistic factors, it is likely that cognitive (e.g., attention and memory) and perceptual (e.g., temporal processing) skills play a key role in modulating speech perception outcomes in this population (Mattys et al., 2025). Future research could help to tease apart the relative importance of these factors in determining downstream effects on speech-perception outcomes.

## Conclusions

The current study was the first systematic examination of listening-related fatigue in L2 listeners. We provide evidence that some L2 listeners experience heightened listening-related fatigue in their daily life, and that these experiences are driven primarily by language proficiency. Results also support the notion that, until an individual reaches high levels of L2 proficiency, increased usage of a second language may come at the cost of increased listening-related fatigue.

## Supplementary Information

Below is the link to the electronic supplementary material.

Supplementary file1 (DOCX 17 kb)

## Data Availability

Raw and summary data can be accessed from our Open Science Framework (OSF) project homepage (https://osf.io/wj59n/overview).

## Footnotes

1 See also the literature on the ‘linguistic similarity hypothesis’ which makes a case for potential negative effects of L1/L2 linguistic similarity on speech-perception outcomes (e.g., Bradlow et al., 2010).

2 Due to an unforeseen error, the attention check was not programmed for the first 62 participants in the study, all of whom were non-IE L2-English listeners. We kept these participants in our analysis as there was no indication in the study data that these participants were not paying attention.

3 In this formula, we estimate that responding ‘mostly L1’ (2) means that participants use L1 approximately 75% of the time, and therefore L2 approximately 25% of time, and vice versa for ‘mostly L2’ (4) responses.

4 In a revised regression model that included only self-rated L2 proficiency and LexTALE vocabulary as predictors, both still contributed unique individual variance in listening-related fatigue (*p*s ≤.004).

## Declarations

### Open practices statement

Methodological plans and hypotheses for this study were pre-registered (https://osf.io/wj59n/registrations). Raw and summary data can be accessed from our Open Science Framework (OSF) project homepage (https://osf.io/wj59n/overview).
