# Supplementary material for: Language proficiency drives listening-related fatigue in those who communicate in English as their second language
Source: Psychon Bull Rev. 2026 Sep 17;33(7):239. doi: 10.3758/s13423-026-03005-3 (PMC13585771; doi:10.3758/s13423-026-03005-3)
Supplement: Supplementary file 1 [file 13423_2026_3005_MOESM1_ESM.docx]

**Deviations from preregistration**

- Norwegian included in list of IE languages, replacing Spanish, to avoid recruiting members of the Spanish-English US population with unusually high L2-English proficiency.
- We included an attention check in the Vanderbilt Fatigue Scale, which all participants completed except the first 62 participants as the decision to include this was taken shortly after data collection began.
- Three participants were removed for completing the experiment exceptionally fast (< 25% of median completion time) and/or providing uninterpretable responses (e.g., responding with a number to a question asking them to report their ‘Language A’).
- One participant was removed for extremely poor performance on the LexTALE task (scoring just 6% accuracy, 6.26 SDs below the mean) suggesting either inattention or very low English proficiency.
- Due to high correlations (*r*s ≥ .8) between these predictor variables, formal language use and dense code-switching variables were removed from the multiple regression analysis.
